# Overwintering Hosts for the Exotic Leafroller Parasitoid, *Colpoclypeus florus*: Implications for Habitat Manipulation to Augment Biological Control of Leafrollers in Pome Fruits

**DOI:** 10.1673/031.010.7501

**Published:** 2010-06-25

**Authors:** R. S. Pfannenstiel, T. R. Unruh, J. F. Brunner

**Affiliations:** ^1^ Tree Fruit Research and Extension Center, Washington State University, 1100 N. Western Ave, Wenatchee, WA 98801; ^2^ USDA-ARS, 5230 Konnowac Pass Rd, Wapato, WA 98951; ^3^ USDA-ARS, 2413 E. Hwy 83, Weslaco, TX 78596

**Keywords:** alternate host, *Ancylis comptana*, diapause

## Abstract

Thirty sites of managed and native habitats were surveyed for leafrollers (Lepidoptera: Tortricidae) in the apple producing region of central Washington State and northern Oregon from September through November 1997–2000 to discover species that supported overwintering by the parasitoid *Colpoclypeus florus* (Walker) (Hymenoptera: Eulophidae). *C. florus,* a species introduced from Europe, requires medium to large host larvae late in autumn on which to overwinter, and few leafroller species display this biology. Over the four years, five potential *C*. *florus* hosts were collected, including: *Ancylis comptana* (Froelich), *Xenotemna pallorana* (Robinson), and *Syndemis* sp. (Tortricidae), *Filatima* sp. (Gelechiidae), and *Caloptilia burgessiellia* (Zeller) (Gracillariidae). Of these, *A. comptana, Syndemis* sp., and *Filatima* sp. have been confirmed as overwintering hosts for *C*. *florus.* During the four years, the *Syndemis* sp. was rare and observed at only one location feeding on redosier dogwood, *Cornus sericea* L. (Cornales: Cornaceae) although, at this location, many of the larvae collected were parasitized by *C. florus. Filatima* sp. was common in the Yakima valley feeding on balsam poplar, *Populus balsamifera* L. ssp. *trichocarpa* (Torr. & Gray ex Hook) Brayshaw (Malpighiales: Salicaceae) but was rarely parasitized. *A. comptana,* however, was collected at many locations in central Washington and was frequently found as an overwintering host for *C. florus. A. comptana* was found feeding on two Rosaceae: Wood's rose, *Rosa woodsii* Lindl., and strawberry, *Fragaria ananassa* Duchesne (Rosales: Rosaceae). Based on the number of host larvae collected, *A. comptana* appears to be the primary overwintering host for *C*. *florus* in Washington. Introduction of *A. comptana* populations to near-orchard habitats may facilitate biological control of leafrollers that are orchard pests.

## Introduction

The leafrollers (Lepidoptera: Tortricidae) *Choristoneura rosaceana* and *Pandemis pyrusana* are important pests of pome fruits, especially apples, in Central Washington (Beers et al. 1993). Leafrollers have become common pests in apples, particularly in orchards that are using bio-rational control programs for the codling moth *Cydia pomonella* (Gut and Brunner 1998). In 1992, a European parasitoid of leafrollers, *Colpoclypeus florus* Walker (Hymenoptera: Eulophidae), was discovered attacking leafrollers in unsprayed apple orchards of central Washington (Brunner 1996). The population in Washington may stem from introduction into Ontario, Canada in the 1960s (Brunner 1996) or from a separate unintentional introduction. Since 1992, this parasitoid has been found from northeastern Oregon and the Yakima valley in Washington (Pfannenstiel et al. 2004; and unpublished data) north into the Okanagan valley in British Columbia, Canada (Vakenti et al. 2001; Cossentine et al. 2004). *C*. *florus* is the dominant parasitoid of leafrollers in Europe (Evenhuis 1974; Gruys 1982; Evenhuis and Vlug 1983; Gruys and Vaal 1984) and has become a dominant parasitoid of leafrollers, particularly *C. rosaceana* and *P. pyrusana*, in Washington (Brunner 1996; Pfannenstiel et al. 1996, 2000).

*C*. *florus* has been reported to overwinter in the larval or pupal stage on suitably sized leafroller hosts (Gruys and Vaal 1982) but observations in Washington suggested that it overwinters only in the larval stage (Pfannenstiel and Unruh, personal observations). *C*. *florus* attacks medium to large larvae (Dijkstra 1986; van Veen and van Wijk 1987), and for the leafrollers that are pests of Washington apples, this would correspond to the 4^th^ and 5^th^ instars. The leafrollers that are present in orchards in central Washington overwinter as 1^st^ and 2^nd^ instars, (*C*. *rosaceana* and *P. pyrusana*) or as eggs (*Archips rosanus*) (Beers 1993) and would not be suitable overwintering hosts (Pfannenstiel et al. 2000). *C*. *florus* must find suitable hosts for overwintering outside of orchards. Because *C. florus* must leave the orchards in the fall, there is a lack of congruence in spring between the *C*. *florus* emerging outside orchards and leafrollers developing in orchards. Hence, in the spring, emerging *C*. *florus* populations may occur primarily in isolated overwintering habitats distant from orchards leading to low levels of parasitism in orchards, particularly in the spring leafroller generation (Gruys 1982; Brunner 1996).

It has been hypothesized both for Europe and Washington that the low availability of alternate host larvae in the fall near orchards causes *C*. *florus* populations to seasonally disappear from orchards (Gruys 1982; Brunner 1996; Pfannenstiel et al. 2000; Pfannenstiel and Unruh 2004). Attempts to identify overwintering hosts in much of Europe have been unsuccessful (Evenhuis and Vlug 1983). However, overwintering has been reported by Karczewski (1962) in Poland and by van Veen and van Wijk (1987) in Italy on several tortricid hosts. In only one instance has significant spring parasitism of leafrollers by *C*. *florus* been reported; this was in association with habitats containing abundant overwintering hosts (Karczewski 1962). *C. florus* does not overwinter on one of its primary orchard hosts *Adoxophyes orana* in the Netherlands, where *A. orana* overwinters as a 2^nd^ instar (Gruys 1982). van Veen and van Wijk (1987) found 30% parasitism of diapausing *A. orana* during the autumn in northern Italy where it apparently overwinters as a 3^rd^ instar, however, they did not report whether the *C*. *florus* was in diapause.

Despite the inability to locate overwintering hosts in the Netherlands, diapausing *C. florus* could easily be obtained by placing laboratory reared suitable hosts into apple orchards in autumn in the Netherlands (Dijkstra 1986). Similarly, diapausing *C*. *florus* were easily obtained in Washington through the placement of sentinel leafroller larvae in apple orchards during the fall (Pfannenstiel et al. 2000). *C*. *florus* females were actively searching in these orchards through October with parasitism of sentinel leafrollers frequently approaching 100% in the fall in some orchards. In contrast, parasitism of naturally occurring or sentinel leafrollers in the orchards in spring was typically low (Brunner 1996; Pfannenstiel et al. 2000) and consistent with the absence of overwintering hosts in or near orchards.

This study was initiated to identify leafrollers that provide overwintering hosts for *C*. *florus* in central Washington. The focus was on locating leafrollers that overwinter as medium to large larvae, the host size suitable for parasitism by *C. florus* (Gruys and Vaal 1984; Dijkstra 1986; van Veen and van Wijk 1987).

## Materials and Methods

Agricultural and non-agricultural habitats in central Washington were sampled for leafrollers during September, October, and November from 1997 to 2000. Most locations were in areas that *C*. *florus* was known to be established (Brunner 1996; Pfannenstiel and Unruh 2004), and most, but not all, habitats sampled were within 5 k of an orchard. The apple producing region in central Washington is east of the Cascade mountain range and is primarily in the rain-shadow of these mountains, receiving 20–30 cm of rainfall per year, much of it in the winter as rain or snow. There are two primary non-orchard habitats in the apple producing areas: 1) the sage, bitterbrush, grassland (shrub-steppe) that consist of short-lived spring annual and perennial cheat and bunch grasses and xeric adapted species including big sage, *Artemisia tridentata* Nutt. (Asterales: Asateraceae) and bitterbrush, *Purshia tridentata* (Pursh) de Canolle (Rosales: Rosaceae) and more lush and diverse riparian habitats dominated by willow (*Salix* spp.) and balsam poplar (*Populus balsamifera* L. ssp. *trichocarpa* (Torr. & Gray ex Hook) Brayshaw (Malpighiales: Salicaceae)) at lower elevations and redosier dogwood, *Cornus sericea* L. (Cornales: Cornaceae) and chokecherry, *Prunus virginiana* L.(Rosales: Rosaceae) at higher elevations. During the summer and fall, much of the vegetation dies, except in riparian areas or among xeric adapted plants such as sage and bitterbrush, or in the weeds along orchard margins where irrigation water may spread. The search was focused in riparian areas, although collections were also done in orchard margins and the shrub-steppe habitats. Additionally, commercial alfalfa fields were sampled specifically for *Xenotemna pallorana* (Robinson) (Lepidoptera: Tortricidae).

At all sites, all plant types were examined for evidence of leafroller infestation and, particularly, the presence of leafroller larvae. In addition to perennial species, the many herbaceous species present including the grasses (Poaceae) were examined. Leafroller infestations were characterized by the presence of foliage that had been rolled or folded and was held in this position by silk. Where evidence of leafroller feeding was observed, a more intensive search was conducted, and leafroller larvae were collected. Samples of > 25 individuals were collected whenever possible; collections of < 25 individuals occurred when that was all that could be located after about 1 h of searching.

In 1997, samples were collected in the Wenatchee area of central Washington, the site of the first discovery of *C*. *florus* in the United States (Brunner 1996). In 1998, the survey was expanded to include the Kittitas and Yakima Valleys and the Columbia Basin in central and southern Washington. The Hood River valley of northern Oregon and the adjacent White Salmon valley in southern Washington, both areas where *C*. *florus* had been released into apple orchards, were also sampled in 1998. In 1999, leafroller sampling emphasized areas with high densities of Wood's Rose, *Rosa woodsii* Lindl. (Rosales: Rosaceae), near apple orchards or feral apples. In 2000, sampling was restricted to a gelechiid associated with balsam poplar near Yakima, WA.

Leafrollers were collected intact within their retreat whenever possible. In 1997, leafrollers were collected into 1 gallon zip-lock bags and placed into a cooler with blue ice packs covered by a towel to buffer the temperature for the larvae. Within 48 h, larvae with their silken retreats were individually placed into 9 × 50 mm Petri dishes (Falcon 6006) with tight fitting lids for rearing at 22° C and a 14:10 L:D photoperiod to evaluate parasitism. When needed, larvae were provided with additional food, which consisted of foliage from the host on which they were collected, or in the case of *X*. *pallorana,* artificial leafroller diet (Shorey and Hale 1965). In 1998 through 2000, leafrollers, while in their retreats, were collected directly into the 9 × 50 mm Petri dishes and placed into wood Stevenson screen weather enclosures to allow exposure to winter conditions. Samples were checked in late November to discover any moths or parasitoids that emerged prior to overwintering. Emergence of overwintering insects was evaluated during the following spring beginning in early April of 1999 and 2000. Samples collected in 2000 were evaluated in June 2001. Where possible, leafroller larvae were visually identified on collection or before placement in overwintering enclosures. All adults were identified on emergence or curated for later identification. Identification of specific hosts for overwintering *C*. *florus* at each site was done by a combination of identifying larval hosts in the fall and identifying surviving unparasitized individuals that emerged in the spring as moths. During this study, only one species of leafroller was located at any one site on any one plant type.

## Results

**Figure 1.**
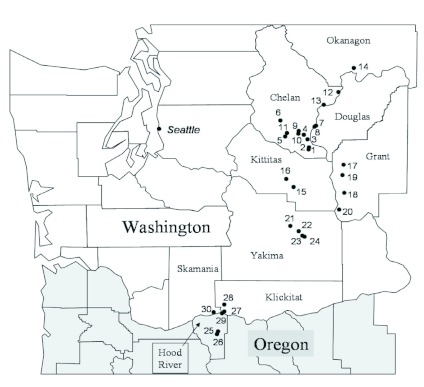
Survey locations in Washington and Oregon, USA, 1997–2000. Site numbers correspond to the map keys in Tables 2, 3. High quality figures are available online.

Leafrollers were collected from 21 of the 30 sites sampled in the fruit producing region of central Washington and northern Oregon (Table 1, Figure 1). Plant communities known to support leafroller development varied considerably from site to site (Table 1). Leafrollers large enough for parasitism by *C*. *florus* were difficult to locate in most habitats in September and October of all years. Other than small diapausing (unsuitable) larvae of *C. rosaceana* and *P. pyrusana,* only five species of potential leafroller hosts were collected after September 1 in any year. These included *Ancylis comptona* (Froelich), *X*. *pallorana,* and *Syndemis* sp. (Tortricidae), *Filatima* sp. (Gelechiidae), and *Caloptilia burgessiellia* (Zeller) (Gracillariidae). *Syndemis* sp. was the only leafroller that was easily recognizable as large enough to support parasitism by *C*. *florus*; the stages of the other four leafrollers all seemed to be smaller than the suitable sizes of leafrollers typically parasitized by *C*. *florus. A. comptana* was the most common of these leafrollers and, once identified, could be collected from roses in numerous locations in the Columbia river basin near Wenatchee and the Kittitas, Wenatchee and Yakima valleys (Site Nos. 1–4, 6–8, 13, 15 & 16; Figure 1; Tables 1, 4).

**Table 1.**
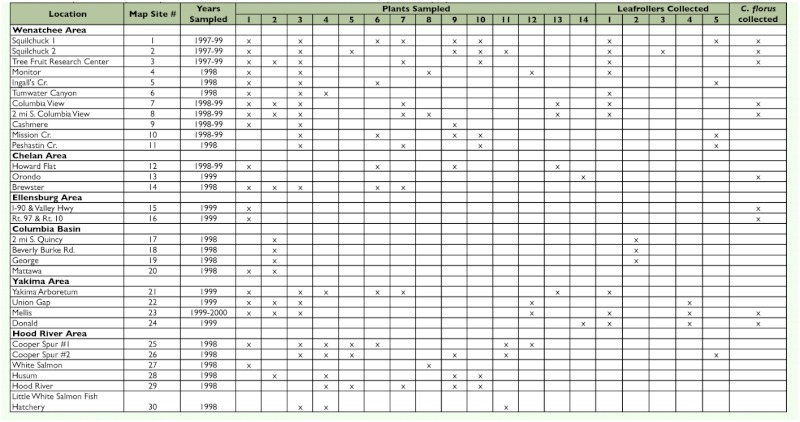
Location characterizations for sites in Washington and Oregon sampled from 1997 to 2000 for diapausing *C*. *florus*. Included is a summary of the leafroller species and the plants they were collected from at each location. Collection of leafrollers parasitized by *C*. *florus* is also shown.

During the three years that roses were included in the survey, *A. comptana* was by far the most frequently collected leafroller (Table 4). Local populations of *A. comptana* in rose patches varied considerably in density from undetectable to multiple individuals found in each rose shoot (terminal) (Pfannenstiel personal observation). *Syndemis* sp. was rare, with only 14 individuals collected over the three years of the survey. All *Syndemis* sp. larvae were collected at one location feeding on redosier dogwood, despite sampling that host plant in numerous locations (Table 1).

*X. pallorana* was only occasionally found in certain alfalfa fields in the Columbia basin in the fall, and only small numbers were collected for determining parasitism. *Filatima* sp. larvae were common in the Yakima valley feeding on balsam poplar, but this host plant was not examined at other survey locations. *C*. *burgessiella* was found in several locations feeding on redosier dogwood but always at very low population densities, so few were collected. This species produces very little silk in comparison to the other leafroller species and is unlikely to be a suitable host for *C*. *florus,* whose parasitism behavior is intimately related to silk production (Dijkstra 1986). When *C*. *florus* attacks a potential host, it first stings the larva in the vicinity of the head and then waits within the larval retreat. The venom injected by the parasitoid causes the larva to spin a dense web of silk that has a different consistency than the normal larval silk. About 12 to 36 hours after attacking the host the *C*. *florus* female lays its eggs directly on the silk, not on the host. When the parasitoid larvae hatch, they crawl along the silk strands until they reach the host and begin feeding externally.

**Table 2.**
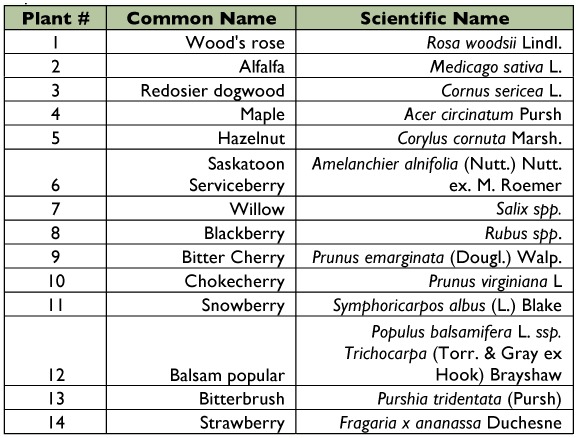
Key to plants sampled at each location.

**Table 3.**
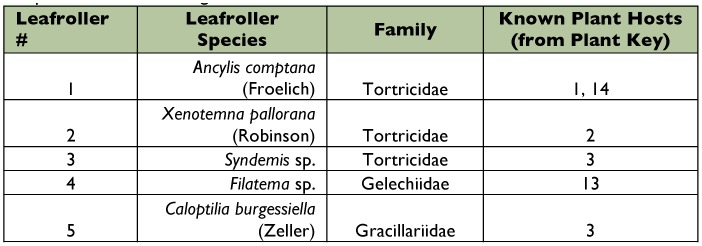
Leafroller species collected during the fall.

Diapausing *C. florus* were first collected during this survey on 19 September 1997 on *Syndemis* sp. larvae feeding on redosier dogwood at the Squilchuck 2 site (Figure 1, Table 5). *C*. *florus* in diapause were also collected on *Syndemis* sp. in the fall of 1998 and 1999 at this location. This leafroller was collected at no other location despite spring, summer, and fall collections from redosier dogwood at > 25 locations in central and south-central Washington and northern Oregon (unpublished data). *C. florus* were also discovered diapausing on *A. comptana* feeding on Wood's rose at Squilchuck 1 in 1997. Additional observations of *C*. *florus* diapausing on *A. comptana* were made on roses at 3 sites in 1998 and 7 sites in 1999 and in strawberries at one site in 1999 (Table 5).

**Table 4.**
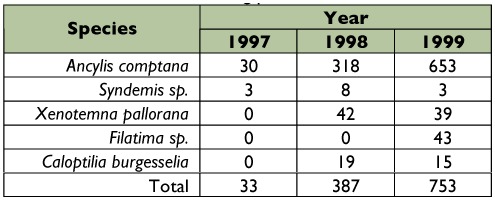
Leafrollers collected for evaluation of overwintering parasitism, 1997–99.

**Table 5.**
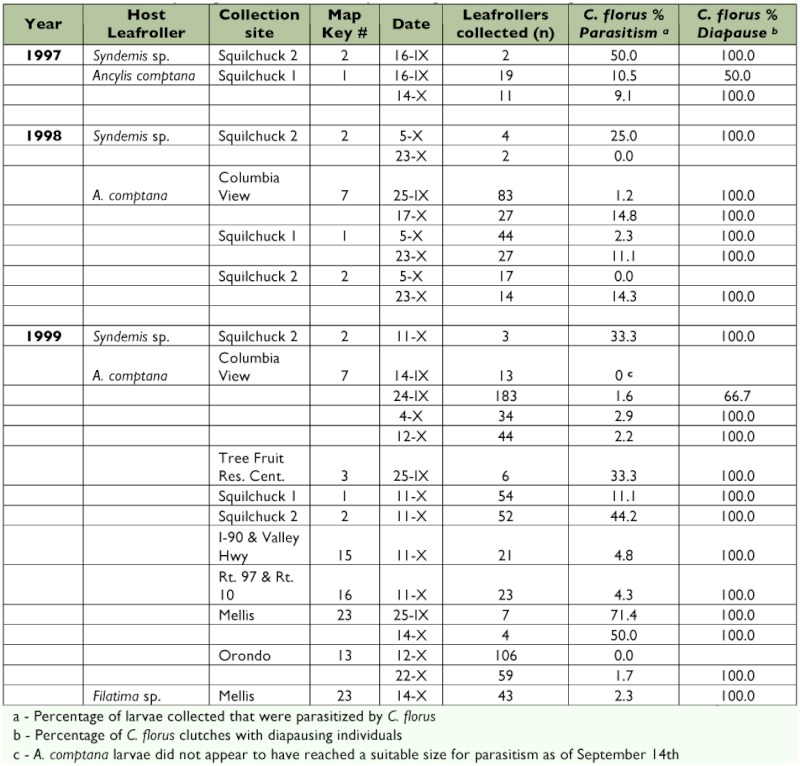
Collection of diapausing C. florus from naturally occurring fall hosts in Washington State.

In 1999, diapausing *C. florus* were observed on single *Filatima* sp., feeding on balsam poplar out of a sample of 43 larvae (2.3%). This was a low level of parasitism of *Filatima* sp. compared to the very high parasitism of sentinel *P. pyrusana* (100%) in nearby apples and parasitism of *A. comptona* (71%, *n* = 7) in an adjacent rose patch. The small number of *A. comptana* collected for this sample was due to its scarcity in this area. Many other rose patches were sampled in this general area (1 km in either direction along the north bank of the Yakima River) during that day, and no other *A. comptana* were collected, nor was additional evidence of larval feeding observed. In 2000, > 200 *Filatima* sp. were collected, and no parasitism by *C*. *florus* was observed, despite very high parasitism of sentinel *P. pyrusana* (> 95%) in nearby orchards. No parasitism of *X*. *pallorana* or *C*. *burgesselia* by *C. florus* was observed during this survey.

Three of the five leafroller species, *A. comptana, Syndemis* sp., and *Filatima* sp., that were observed to overwinter in potentially suitable stages for *C*. *florus* were observed with diapausing *C*. *florus* in the field. However, only *A. comptana* appeared to be a numerically important overwintering host. Parasitism of *A. comptana* by *C*. *florus* varied widely from site to site ranging from 0 to 44% (sites with *n* > 10; up to 71% with smaller sample sizes) (Table 5).

*Syndemis* sp. was only observed at one location and in small numbers, thus this host is unlikely to play an important role in the population dynamics of *C*. *florus.* However, despite its rarity, *C*. *florus* larvae diapausing on *Syndemis* sp. were collected each fall. Parasitism of *Filatima* sp. was much more difficult to observe. Despite the ease of locating larvae of this species, *Filatima* sp. appears to not be a particularly suitable host for *C. florus.* It is smaller than *A. comptana* and may rarely reach a size large enough for *C. florus* to parasitize. *C*. *burgessiellia* does not appear to be a suitable host for *C. florus* as there were no recoveries with parasitism by *C*. *florus.* The third tortricid collected, *X*. *pallorana*, is very suitable for parasitism by *C*. *florus* (Nobbs 1998), but it was not collected in areas where *C*. *florus* was common. No parasitism was observed in the field. *X*. *pallorana* also appears to overwinter as relatively small larva (2^nd^ to 3^rd^ instars), and its size appears too small to support overwintering by *C*. *florus.* However, the overwintering instars of *X*. *pallorana* have not been definitively determined.

## Discussion

*C*. *florus* has been reported to be the most important parasitoid of leafrollers in Europe (Evenhuis 1974; Evenhuis and Vlug 1983). Although it was noted that *C*. *florus* overwintered outside of orchards, in only a few cases have overwintering hosts been observed in Europe (Karczewski 1962; van Veen and van Wijk 1987). Other researchers searched for hosts in the Netherlands for extended periods without locating an overwintering host (Evenhuis and Vlug 1983).

Identification of the common overwintering host for *C. florus* in Washington may allow manipulation of *C*. *florus* populations in and around apple orchards. Few orchards in central Washington appear to be in close proximity to *C*. *florus* overwintering sites, and this is believed to be responsible for inhibiting successful biological control of orchard leafroller pests (Pfannenstiel et al. 2000; Pfannenstiel and Unruh 2004). A common pattern of parasitism in both Washington State and Europe is for the proportion of hosts parasitized to be low, typically < 5%, during the spring leafroller generations (most univoltine leafrollers and the 1st generation of multivoltine species) and then high during the summer generations, frequently > 50% (Gruys 1982; Gruys and Vaal 1984; Pfannenstiel et al. 2000; Pfannenstiel and Unruh 2004). The low spring parasitism may be due to the spatial separation between orchards and *C*. *florus* overwintering sites. Overcoming the paucity of hosts for *C. florus* in or near orchards in the fall by creating habitats with suitable overwintering hosts may result in higher spring parasitism of orchard leafrollers, leading to successful biological control.

The most abundant overwintering host for *C*. *florus* in central Washington observed during this study was *A. comptana*; it was common in the Columbia valley near Wenatchee, WA, the Kittitas valley and the Wenatchee river valley, and was less common in the Yakima valley (Figure 1, Table 1). *C. florus* females were frequently observed searching for hosts in the fall at numerous locations throughout the study area. The latest that a female was observed searching was November 1, by which time temperatures have cooled significantly (mean low temperature in Wenatchee as of November 1 was about 2° C). A host of *C*. *florus* in Europe (Gambaro 1971; Monta et al. 1973), *A. comptana* invaded North America in the mid 1800's (Walsh and Riley 1869) and is found throughout much of the United States, where it is currently a sporadic pest of strawberries (Schaefers and Schanks 1991).

The impact of an alternate host on a host species that shares a parasitoid sp. has been termed “apparent competition” (Holt and Lawton 1993, 1994). Apparent competition may affect biological control of *C*. *rosaceana* and *P. pyrusana* in Washington apple orchards. Having identified a suitable alternate host for *C. florus* in *A. comptana*, populations of it will be introduced near orchard habitats to increase parasitism by *C*. *florus*, particularly in the spring. Although the theory associated with apparent competition has been thoroughly developed, few good examples of its impact in parasitoid-host systems have been observed (Chaneton and Bonsall 2000). The system where this has been documented most thoroughly is the leafhopper (*Erythroneura elegantula*)-*Anagrus* spp. system in California vineyards. Habitats containing alternate hosts for overwintering by *Anagrus* spp., including blackberry patches and prune plantings that are in proximity to vineyards can lead to reduction of pest leafhopper populations in grapes (Settle and Wilson 1990, Corbet and Rosenheim 1996). However, biological control of leafhoppers in this system has been inconsistent, most likely because the populations of alternate hosts in the relatively small overwintering refuges are decimated by populations of *Anagrus* spp. emigrating from vineyards in the fall (Daane and Costello 2002). In many ways, the leafhopper-*Anagrus* system in California resembles the leafroller-*C*. *florus* system in Washington pome fruits. Parasitoids must leave the crop to overwinter on alternate hosts in distant or small refuges, but do not return in either a high enough density or early enough in the season for adequate biological control.

When hosts share a parasitoid that causes significant mortality, local extinction of alternate hosts is an important concern (Holt and Lawton 1993, 1994). One of the major concerns in establishing populations of *A. comptana* for augmentation of *C. florus* is that the populations will need to be large enough to avoid local extirpation by the *C. florus* emerging from orchards in the fall. When sentinels of the pest leafrollers are placed into orchards in the fall, parasitism is frequently 100%, so it appears that *C*. *florus* populations may be large enough to extirpate small populations of alternate hosts. One reason that *A. comptana* are so rare at the Mellis site may be because it is adjacent to a large area of orchards and the emigrating *C. florus* severely reduced its population. As attempts are made to establish populations of *A. comptana* near orchards, one advantage may be that its larvae appear to only be suitable in its final instar and will only be pressured by *C*. *florus* for a relatively short period during summer generations. In the fall, *A. comptana* do not become suitable as hosts until well after *C*. *florus* emerges from orchard hosts, so there may be significant parasitoid mortality during this period because of the absence of appropriate hosts.

*A. comptana* has several characteristics that make it a good alternate host for manipulation to augment *C. florus* populations, including a relatively narrow host range including Wood's rose and strawberries, and a host plant, Wood's rose, that is easy to establish and maintain near orchards. In the apple producing region of Washington, there is little production of strawberries; therefore augmentation of *A. comptana* populations would be unlikely to cause significant negative effects on nearby crops. The plant host that would be most likely used for manipulation, *R. woodsii*, is common in this relatively arid region and should survive near orchards with only minor water augmentation. As an additional benefit, *A. comptana* is a host for another occasionally important parasitoid of leafroller, *Oncophanes americanus* (Pfannenstiel unpublished data). Future studies will evaluate the potential of creating near orchard habitats containing roses and strawberries and introducing *A. comptana* to increase the local density of *C*. *florus* and subsequent parasitism of leafrollers in orchards, particularly in the spring.
